# Complex problems require complex solutions: the utility of social quality theory for addressing the Social Determinants of Health

**DOI:** 10.1186/1471-2458-11-630

**Published:** 2011-08-05

**Authors:** Paul R Ward, Samantha B Meyer, Fiona Verity, Tiffany K Gill, Tini CN Luong

**Affiliations:** 1 Discipline of Public Health, Flinders University, Sturt Road, Bedford Park, Adelaide, 5042, Australia; 2 Department of Social Work and Social Planning, Flinders University, Sturt Road, Bedford Park, Adelaide, 5042, Australia; 3 Population Research & Outcome Studies, Discipline of Medicine, University of Adelaide, 122 Frome Street, Adelaide, 5000, Australia

## Abstract

**Background:**

In order to improve the health of the most vulnerable groups in society, the WHO Commission on Social Determinants of Health (CSDH) called for multi-sectoral action, which requires research and policy on the multiple and inter-linking factors shaping health outcomes. Most conceptual tools available to researchers tend to focus on singular and specific social determinants of health (SDH) (e.g. social capital, empowerment, social inclusion). However, a new and innovative conceptual framework, known as social quality theory, facilitates a more complex and complete understanding of the SDH, with its focus on four domains: social cohesion, social inclusion, social empowerment and socioeconomic security, all within the same conceptual framework. This paper provides both an overview of social quality theory in addition to findings from a national survey of social quality in Australia, as a means of demonstrating the operationalisation of the theory.

**Methods:**

Data were collected using a national random postal survey of 1044 respondents in September, 2009. Multivariate logistic regression analysis was conducted.

**Results:**

Statistical analysis revealed that people on lower incomes (less than $45000) experience worse social quality across all of the four domains: lower socio-economic security, lower levels of membership of organisations (lower social cohesion), higher levels of discrimination and less political action (lower social inclusion) and lower social empowerment. The findings were mixed in terms of age, with people over 65 years experiencing lower socio-economic security, but having higher levels of social cohesion, experiencing lower levels of discrimination (higher social inclusion) and engaging in more political action (higher social empowerment). In terms of gender, women had higher social cohesion than men, although also experienced more discrimination (lower social inclusion).

**Conclusions:**

Applying social quality theory allows researchers and policy makers to measure and respond to the multiple sources of oppression and advantage experienced by certain population groups, and to monitor the effectiveness of interventions over time.

## Background

Both trans-nationally [1-3] and within particular countries [4], it is widely recognised that public health policy and practice needs to focus on addressing the Social Determinants of Health (SDH) in order to increase the health of the most vulnerable and disadvantaged groups. By focussing on developing relevant and appropriate policy and practice responses for such groups, it is hoped that we can redress the current inequities in health outcomes between the most and least advantaged groups within society. The Commission on the Social Determinants of Health (CSDH) recognised the multiple forms of oppression and disadvantage experienced by the poorest members of society [1]. Building on seminal multi-national agreements such as the Ottawa Charter [5], the Alma Ata Declaration [6] and the Bangkok Declaration [7], the CSDH called for a 'joined up', multi-sectoral approach to addressing the problem.

We concur with the need to focus on both the multiple forms of disadvantage and thus the complex and holistic policy responses required, although we argue that many conceptual frameworks currently used in public health research do not lend themselves easily to being useful for these purposes. For example, there are large amounts of research which provide evidence that certain population groups are more socially excluded [8], have lower levels of social capital [9], have poorer access to financial resources, health promoting or curative services [10] and that some groups are disempowered [11]. All of these factors have been shown to be SDH, in that higher levels of social inclusion, social capital, access to finance and services and empowerment are all 'good for your health'; however, taken on their own, these studies are useful only in so far as they paint part of the picture as to both the problems and solutions for increasing the health of such groups. What they do not do is provide both a conceptual and methodological framework for linking these various concepts for the same population groups, which would then highlight the potentially multiple 'problems' that certain population groups encounter, or the particular 'problems' that other groups encounter. Research studies may highlight the need to implement policy to increase the social capital for particular groups, or to facilitate more socially inclusive policies or systems, but rarely can such studies (due to their conceptual limitations) provide evidence for policies and systems which attend to the multiplicity of needs highlighted by the CSDH.

This paper attempts to redress this problem. Firstly we introduce a theoretical and conceptual framework developed in social policy in Europe, known as social quality theory [12-16], which aims to overcome the 'silo' problem mentioned above and provides a holistic approach to understanding social problems and potentials for social change. Secondly, we go on to describe a study of social quality in Australia, and in so doing, highlight the utility of such an approach for researchers and policy makers in public health interested in both understanding and responding to the SDH for the most vulnerable groups in society.

### Background to social quality theory

The notion of social quality is gaining international recognition as an innovative theoretical and methodological tool for researchers and policy makers in social policy [12,14-22]. However, scant attention has been given to the utility of social quality theory to public health research and policy, which requires remedy given its obvious links to the SDH.

Social quality has been defined by Beck (1998: 3) as *"the extent to which people are able to participate in the social, economic life and development of their communities under conditions which enhance their wellbeing and individual potential*" [22]. Social quality theory was initially developed by the European Network Indicators of Social Quality (ENISQ). The ENISQ undertook substantial research in order to develop the conceptual and methodological tools to measure social quality, including a detailed process of development, construction and validity of the indicators and domains [23]. The indicators (or metrics) of social quality were specifically developed so that governments and researchers could assess social quality within and between societies or Nation States, using only routinely available data sources [24]. Whilst this has tremendous benefits in terms of not needing to design and implement primary research, it also relies on existing datasets, which are often collected for administrative purposes and are often relatively old. Therefore, we used the indicators to develop a new social quality questionnaire to measure social quality in Australia. In this way, we have advanced the methodological and practical aspects of social quality theory by providing researchers and policy makers with a readily available instrument to measure social quality in their jurisdictions. Until now, there had been no studies, either within or outside Europe, which had undertaken primary data collection on social quality.

Social quality theory was originally developed as a response to the hegemony of individualised quality of life measures [13], and indeed the relative demise of notions of the 'social' within the social sciences during the move to post modernity [25]. Walker (2009: 214) argues that contemporary Western societies are preoccupied with measuring and increasing our well-being, quality of life, happiness and so on *as individuals*, rather than as individuals in groups, communities and other social relations. The development of social quality theory is an attempt to redress this imbalance, by refocusing on 'the social', which does not sit in contradistinction to 'the individual', but rather, akin to the ideas of Bhaskar [26], Archer [27] and Giddens [28,29], are part and parcel of the same phenomena.

Social quality theory does not dismiss the individual quality of life approach, since it is useful for clinical situations and individualised solutions. However, the point is that it provides relatively little use for developing population-level social or public health policy. The individual quality of life approach can tell us a great deal about how to improve individual circumstances (e.g. functional wellbeing, psychological needs, cognitive impairments etc.) but it cannot elucidate either the reasons why some population groups fair worse than others in society, or more importantly, how we may be able to respond in terms of policy and practice [12]. In addition, individual perspectives on quality of life tend to avoid consideration of the involvement of political and normative factors [12].

Social quality theory has both ideological and methodological underpinnings. In terms of its underlying ideology, social quality theory argues that there are four key *normative factors *that determine the quality of the social structures, policies and relationships within a society: social justice; solidarity; equal value of all humans; and human dignity [30]. A society can be judged according to these normative factors, both in a global sense (i.e. how good is the social quality of a particular society) but also in terms of the specific normative factors (i.e. which factors require policy response in a particular society). However, on their own, these normative factors are not easily operationalised and do not have a methodological framework. Therefore, within social quality theory, there are a set of *conditional factors *which are aimed at rendering the normative factors 'researchable'. The four conditional factors are socio-economic security (linked to social justice), social cohesion (linked to solidarity), social inclusion (linked to equal value) and social empowerment (linked to human dignity).

Socio-economic security is concerned with the extent to which people or groups have access to, utilisation of, and successful outcomes related to, a variety of resources over time, and the protection from poverty and other forms of material deprivation. These resources may be associated with, among other things, finance, housing, healthcare, employment and education, which have been shown to be important in shaping inequities in health and health care [1,31]. Social cohesion relates to the extent to which people and groups share social norms and values (and is broader than the popular notion of social capital), and to issues of solidarity and trust, which are again, particularly important in terms of public health [32]. Social inclusion (or at least, the minimisation of social exclusion) is the extent to which people and groups have access to and are integrated into the different institutions and social relations of 'everyday life' and the extent to which people and groups 'feel part of'' or included in society at an everyday level. Social empowerment is the extent to which the personal capabilities of individual people are enhanced by social relations. This domain focuses on the enabling factors which empower people to act as social agents [33,34] and fully and meaningfully participate in society [35,36].

As can be seen in this brief overview, the multi-dimensional and multi-level approach represents an advancement of public health research, theory, policy and practice, which is not solely aimed at either individuals or systems, but instead realises the intimate linkages between systems and individuals and thus provides an understanding of both within the same theoretical framework. The long-term aim of developing and implementing social quality theory is to enhance the social quality of peoples' lives (especially vulnerable groups), but as already stated, we firstly need to have empirical data on the domains of social quality (and the groups who have lower social quality) before we can inform changes in policy and/or practice.

## Methods

It has been suggested that research in social epidemiology and public health in general is strongly focused on empirical research and remains atheoretical [37]. Richter (2010: 457) suggests "it is very obvious that the status quo in research on social determinants of health needs a change to a stronger accentuation of explanatory approaches." Therefore, operationalising and applying social quality theory provides a theoretical platform from which we can investigate specific areas for policy and practice intervention.

The original indicators of social quality, developed by the ENISQ, were developed into a questionnaire, which was tested for both validity and reliability [20], including collaboration and agreement with the originators of the social quality indicators [38]. The questionnaire had 50 questions, divided into the four conditional factors: 4 questions related to socio-economic security, 11 questions related to social inclusion, 5 questions related to social cohesion, 19 questions related to social empowerment, and 11 demographic questions (the full questionnaire is available on-line - see Additional File 1).

A postal questionnaire survey of a random sample of households was undertaken for each Australian state. It was necessary to divide the national population by state [39] because this study was a national sample and some states contain more residents than others. Therefore, more surveys were sent out to states with higher population numbers (New South Wales 1650, Northern Territory 45, Queensland 971, South Australia 389, Tasmania 120, Victoria 1253, Western Australia 490, Australian Capital Territory 82). The sampling frame was the electronic white pages, which contains postal addresses for all households with a telephone listed. Therefore, a small proportion of households who either do not have a telephone or have "silent" numbers were excluded. However, this possible limitation is outweighed by the fact that the electronic white pages is one of the only representative sources from which a national random sample of postal addresses can be generated.

A copy of the questionnaire, a letter of information, a letter of introduction, and a stamped return envelope was sent to each mail-out address September 2009. A postcard reminder was only sent out to those who had not returned the questionnaire after two weeks.

The hypothesised response rate was around 20% (based on the experience of the research team of conducting similar surveys in Australia), and in order to obtain a final sample size of 1000, it was estimated that an initial sample of 5000 addresses was required. Out of the 5000 surveys that were sent out, 638 were returned due to invalid addresses and 1044 were returned completed surveys. The actual response rate of 24% (1044/4362) was regarded as acceptable for this type of survey because of the decline in participation in survey research. Between 1986-1995, survey response rates in Australia remained consistent at around 50% [40]. However in the past decade, survey response rates have been declining as people become more active in protecting their privacy [41,42]. In addition, the growth of telemarketing may have disillusioned the community and diminished the success of legitimate survey-based social science research. In the absence of a higher response rate, we have documented our efforts to increase the response rate [40]. As noted earlier, reminders postcards were sent to non-responders to ensure as high a response rate as possible [43]. Nevertheless, the potential for survey non-response bias is acknowledged.

After data entry had been completed, an extra two variables were created from the postcode of the respondent. Both variables are derived from the national census. The first variable is called the Socio-Economic Indicator For Areas (or SEIFA) and provides a score for the level of socio-economic deprivation or affluence of the area. The second variable is called Accessibility and Remoteness Indicator for Areas (or ARIA) which provides a score for the distance of the postcode from major service centres. Both of these variables were thought to be potentially important when analysing differences in social quality.

Initially, descriptive analyses were undertaken in order to explore overall levels of social quality. We then performed bivariate logistic regression analyses in order to explore simple associations between a range of socio-demographic variables and the indicators of social quality. For the regression models, four questions identified by the ENISQ in 2004 [38] as indicators of the four domains of social quality were used as dependent variables (i.e. one variable per domain of social quality). The complete questionnaire contained many indicators of social quality that have all been shown to be valid proxies for their relevant social quality domain [20]. All of these variables were found to have statistical significance with the listed demographic variables. For example, questions such as *'Please indicate whether you or your family have experienced any of the following negative life events in the last 12 months?', 'How much do you trust various groups of people?' *were found to be associated with demographic variables; however, for the purpose of this paper we have limited our results to one variable per domain. The independent variables chosen to investigate associations between social quality and demographic variables were age, sex, SEIFA IRSD (Socio-economic Index for Areas Index of Relative Socio-economic Disadvantage), ARIA (Accessibility/Remoteness Index of Australia), employment status and income.

Bivariate analyses were conducted using Chi Squares (Cramer's V and Phi) as well as T-tests, one-way ANOVAs, Mann-Whitney U, and Kruskal-Wallis H. Each test produced a table which was subsequently analysed for statistically significant associations. Any bivariate odds ratios with P < 0.25 were then included in multivariate logistic regression analyses [44]. The tables presented in our result section include only the results of bivariate analyses found to have a p value of <0.25. All models were checked for collinearity and goodness of fit [44]. During the bivariate analysis, some of the data were found to have expected cell counts less than five. As a result, many of the categories within the independent variables were collapsed in order to help the data meet the assumption. This was done by recoding the variables using SPSS. Data that could not be collapsed to help meet the assumption have not been included in the results section.

This study was given ethical clearance by the Social and Behavioural Research Ethics Committee at Flinders University.

The following section provides statistical description and analysis of the data. One survey question is presented to investigate each of the four domains of social quality. The sample questions used to investigate each of the four domains were chosen because each question has been identified as an valid and reliable indicator of one of the four domains by the ENISQ in 2004 [38].

## Results

This section of the paper provides statistical description and analysis of the data, focussing specifically on the four conditional factors within the social quality theory, namely socio-economic security, social cohesion, social inclusion and social empowerment. One multivariate regression model is presented for each of the four domains as a means of introducing the practical application of social quality theory. Each of the four models includes one social quality variable (social inclusion, social cohesion etc.) as the dependent variable with the socio-demographic variables (sex, age, income etc.) as independent variables.

### Socio-economic security

There were a number of variables that related to socio-economic security within the dataset, but for the purpose of this paper, we have just used one variable. The question used to measure socio-economic security in the survey is outlined below:

During the past year, did you

1. Save money

2. Just get by

3. Spent some savings

4. Spent savings and borrowed money

In terms of saving or spending money, Table 1 shows a multivariate analysis of the data. Overall, over two thirds of the sample managed to save money or 'just get by', with only one third having to spend savings and/or borrow money. The variable was recoded into two categories, those that just get by or better (68.7%) and those that spent savings or spent and borrow money (31.3%). Univariate odds ratios examined the relationship between those who spent savings or spent and borrowed money and demographic characteristics (age, sex, marital status, work status, income, SEIFA and ARIA), and those with P < 0.25 were entered into a multivariate analysis (Table 1). As can be seen, the only variable left in the model was 'employment status', with retired people being twice as likely than people working to have spent money rather than saved. This highlights the reduced socio-economic security of retired people compared to those working.

**Table 1:** Multivariate odds ratios of demographic factors associated with those who spent money (Socio-economic Security)

	OR	p value
**Employment status**		

Work full time or self employed	1.00	

Work part time	1.19 (0.78-1.80)	0.426

Work without pay, unemployed, student, disability, other	1.58 (0.98-2.55)	0.063

Retired	2.19 (1.58-3.03)	**< 0.001**

Household duties	1.39 (0.73-2.67)	0.321

### Social Cohesion

The variable chosen to examine social cohesion was:

For each of the following organisations, please indicate your membership status

1. Church or religious organisation

2. Sport or recreational organisation

3. Art, music, educational, or cultural organisation

4. Other community based organisation

In terms of membership of organisations, 26% were members of church organisations, 41% were members of sporting organisations, 22% were members of art/cultural organisations and 36% were members of community based organisations.

The variable was then recoded into two categories, those who were a member of at least one organisation (71.0%) and those who were not a member (29.0%). Univariate odds ratios examined the relationship between those who were a member and demographic characteristics (age, sex, marital status, work status, income, SEIFA IRSD and ARIA), and the multivariate analysis is presented in Table 2.

**Table 2:** Multivariate odds ratios of demographic factors associated with membership of organisation(s) (Social Cohesion)

	OR	p value
Age		

18-34 years	1.00	

35-44 years	2.00 (1.11-3.62)	**0.022**

45-54 years	1.60 (0.94-2.72)	0.086

55-64 years	3.08 (1.72-5.53)	**<0.001**

65-74 years	4.90 (2.32-10.37)	**<0.001**

75 years and over	5.18 (2.09-12.82)	**<0.001**

**Income (financial year)**		

$105000-$150000+	1.00	

$45000-$104999	0.59 (0.39-0.88)	**0.011**

-$44999	0.40 (0.25-0.64)	**<0.001**

**Employment status**		

Work full time or self employed	1.00	

Work part time	1.84 (1.14-2.97)	**0.013**

Work without pay, unemployed, student, disability, other	1.74 (0.96-3.17)	0.069

Retired	1.49 (0.82-2.71)	0.195

Household duties	1.51 (0.72-3.16)	0.278

Model stable, Hosmer and Lemeshow, Chi square 4.78, p = 0.783

Table 2 shows people aged over 75 being 5 times more likely to be members of organisations than people aged 18-34 years. Additionally, as income decreases, respondent's level of membership with organisations also decreases. People earning up to $45000 are less than half as likely as those earning over $105000 to be members of organisations. Also, people who work part-time are more likely to be members of organisations than people who work full-time.

### Social Inclusion

Social inclusion deals with an individual's accessibility to institutions and the degree of social integration that the individual attains to [45]. The variable chosen to examine social cohesion was:

During the past 12 months, have you ever experienced discrimination against you due to any of the following reasons?

1. Physical/mental disability

2. Age

3. Sexual harassment

4 Gender

5. Nationality

6. Physical appearance

7. Ethnic background

8. Criminal record

9. Religion

10. Other

The proportion of respondents who had experienced discrimination varied: 4% experienced disability discrimination, 14% age discrimination, 2% sexual discrimination, 7% gender discrimination, 4% nationality discrimination, 6% physical appearance discrimination, 3% ethnic background discrimination, 1% criminal record discrimination, 2% religious discrimination, and 4% other discrimination. The variable was then recoded into two categories, those who had experienced discrimination (23.9%) (excluding the 'other' responses due to the large number of missing) and those who had not experienced discrimination (76.1%). Univariate odds ratios then examined the relationship between those who experienced discrimination and demographic characteristics (age, sex, marital status, work status, income, SEIFA IRSD and ARIA). The multivariate analysis is presented in Table 3.

**Table 3:** Multivariate odds ratios of demographic factors associated with those who experienced discrimination (Social Inclusion)

	OR	p value
**Sex**		

Male	1.00	**0.022**

Female	1.53 (1.06-2.12)	

**Age**		

18-34 years	1.00	

35-44 years	1.16 (0.62-2.17)	0.651

45-54 years	0.64 (0.35-2.17)	0.141

55-64 years	0.54 (0.29-1.03)	0.060

65-74 years	0.54 (0.28-1.08)	0.080

75 years and over	0.43 (0.19-0.98)	**0.044**

**Income (financial year)**		

$105000-$150000+	1.00	

$45000-$104999	1.09 (0.69-1.71)	0.716

-$44999	1.71 (1.04-2.81)	**0.034**

**SEIFA IRSD**		

Lowest quintile	1.00	

Low quintile	0.93 (0.54-1.59)	0.782

Middle quintile	0.81 (0.47-1.41)	0.453

High quintile	0.45 (0.25-0.81)	**0.008**

Highest quintile	0.86 (0.49-1.48)	0.575

Model stable, Hosmer and Lemeshow, Chi square 2.22, p = 0.974

Table 3 shows that women are more likely to experience discrimination than men. In addition, people in the lowest income bracket and people living in areas identified as disadvantaged are most likely to be discriminated against. However, older people are less likely to experience discrimination than younger people.

In interpreting the data in Table 3, we need to be cognisant of the 'blunt' nature of the question on discrimination. For example, the age related finding could potentially be an effect that some older people may be less likely to perceive the same behaviour as discriminatory as when they were younger.

### Social Empowerment

The variable chosen to examine social empowerment was:

Have you or would you participate in any of the political actions listed below?

1. Petition

2. Boycotts

3. Protests

4. Strikes

5. Online political actions

Whilst previous validity testing found this question to be a good proxy for social empowerment, we also recognize the potential for some groups (e.g. wealthier or more powerful groups) to perceive little need to engage in the political actions listed in this question, even though they are highly socially empowered.

In terms of political actions, 70% of people took part in petitions, 19% in boycotts, 23% in protests, 19% in strikes and 13% in online political action.

The variable was then recoded into two categories, those who had participated in a political action (74.1%) and those who had not (25.9%). Univariate odds ratios then examined the relationship between those who participated in a political action and demographic characteristics (age, sex, marital status, work status, income, SEIFA IRSD and ARIA). The multivariate analysis is presented in Table 4.

**Table 4:** Multivariate odds ratios of demographic factors associated with participation in political action (Social Empowerment)

	OR	P value
**Age**		

18-34 years	1.00	

35-44 years	1.60 (0.87-2.94)	0.132

45-54 years	2.20 (1.25-3.90)	**0.007**

55-64 years	2.00 (1.13-3.55)	**0.018**

65-74 years	2.16 (1.18-3.97)	**0.013**

75 years and over	1.21 (0.61-2.39)	0.581

**Income (financial year)**		

$105000-$150000+	1.00	

$45000-$104999	0.79 (0.51-1.21)	0.274

-$44999	0.56 (0.36-0.89)	**0.014**

**ARIA**		

Major cities	1.00	

Inner regional	1.43 (0.54-1.59)	0.080

Outer regional	1.73 (0.47-1.41)	**0.039**

Remote and Very Remote	1.47 (0.52-4.14)	0.464

Model stable, Hosmer and Lemeshow, Chi square 6.16, p = 0.62

Table 4 shows that increasing age was associated with increased political action (until age 75 and over). People on the lowest income level were less likely to get involved in political action although people living in outer regional areas are more likely than people living in major cities to get involved in political actions.

In summary, the findings outlined by the above questions suggest that retired respondents have lower socio-economic security, and women and respondents of lower income have lower social inclusion. However, the findings also suggest lower social quality for disadvantaged individuals who scored poorly in social cohesion, social empowerment and social inclusion. This snapshot of social quality in Australia identifies the gravity of the lack of social quality for poor people, the financial security for older people, and the discrimination experienced by women.

## Discussion

For well over twenty years policy makers, health and social reformers in Australia have advocated the practical sense of an integrated approach in policy making and service delivery; to move from 'silos to a system', towards 'whole of government approaches', and 'health in all' [46-48]. However, an unsettled question remains how this best is done. Our purpose in this paper has been to demonstrate the practical utility of the social quality approach as a means to guide the development of policies, practices and systems in accord with this objective. This utility is argued from the basis of social quality theory as an integrative approach that conceptualises the social as a 'whole'; it draws together theories about social inclusion, social empowerment, social cohesion and socio-economic security in a counterbalance to the hegemony of individual based quality of life measures. It is also an attempt to unify theories and analyses that on their own, concentrate on one aspect of a dynamic and fragmented health and social policy context. Legge et al (1996: 22) describe this context as "...a field of turbulent discourses; different problems, different analyses and different strategies sweeping across the policy field like storm clouds under time lapse photography" [49].

Social quality theory aims to move beyond partial understandings of social problems informed by single disciplinary knowledge, and partial explanations afforded by theories that examine only one area of social life. On the basis of the data reported upon in this paper we contend social quality is useful because of this emphasis on a total or integrated picture. The picture developed in this paper, using the four conditional factors of social quality, is that the social quality of life in Australia is high across the four domains. Socio-economic security is high (except for the third of people who spent savings and/or borrowed money), social cohesion is fairly high (relatively high levels of membership of organisations), social inclusion is high (low levels of perceived discrimination) and social empowerment is fairly high (generally high levels of participation in political actions).

However social quality theory, in drawing together individual measures, does more than provide a holistic picture. It develops a picture of systematic differences in social quality between population groups at a point in time. As seen in this paper, notwithstanding the relatively positive picture of social quality in Australia, there were systematic differences in social quality between population groups. This was most pronounced for people on lower incomes (less than $45000) who were more likely to have spent their savings (lower socio-economic security), had lower levels of membership of organisations (lower social cohesion), experienced higher levels of discrimination (lower social inclusion), and were involved in less political action (lower social empowerment). On all four domains of social quality, people with lower incomes were disadvantaged and may therefore be seen as having generally lower social quality than people on higher incomes. The picture identified higher social quality in older respondents. Older respondents had higher levels of membership of organisations and were more likely to trust (high social cohesion), experienced lower levels of discrimination (high social inclusion) and engaged in more political action (higher social empowerment) than younger respondents. The findings regarding higher levels of membership may be explained by older respondents being more likely to be retired or working part-time and thus, having more opportunity to be members of organisations. The findings also identified lower social quality for older people in respect to financial security. This may be indicative of the fact that older individuals are likely to be living off of pensions and/or retirement plans and are thus, less likely to be saving which does not necessarily indicate poor social quality. However, this finding remains an important consideration in view of the estimations of population numbers who will be over 65 years by the year 2040 and that life expectancy is lengthening. In terms of gender, women experience more discrimination (lower social inclusion) than men. This may reflect lower social inclusion in women but may also be interpreted from the perspective that women and men may differ in their assessment of discriminatory acts.

The difference here between the social quality findings and those from research in the individual research areas (i.e. social cohesion, social inclusion) is that in this study the findings about social cohesion, social empowerment, social inclusion and socio-economic security are pulled together empirically which makes it more amenable to policy changes and action. Our findings highlight areas of lower social quality but in providing a snapshot of the findings, we acknowledge that a more comprehensive presentation of our survey results will lead to more generalisable results regarding areas of high and low social quality. Nonetheless, it is not easy to ignore the gravity of the lack of social quality for poor people, the long term implications of low financial security for older people, or the discrimination experienced by women. Our initial approach to operationalising social quality theory emphasises social solidarity as a policy principle and data analyses may be viewed through a socio-political lens.

In addition there is further practical utility for policy makers derived from value of an integrative perspective on social quality in a context of hyper-fragmentation in the Australian social welfare system. The Australian social policy system has always been a mixed welfare economy [50] but this system is now much more complex and boundaries between sectors further blurred in what Clarke (2004) calls the 'dissolving of the public realm'; privatisation and outsourcing of services to not for profit and profit organizations, and a shift to informal care and market based health and welfare provision [51]. For example, the Australian Bureau of Statistics reported that at the end of June 2007, there were 40,976 not-for-profit organisations in Australia, employing almost 900,000 people [52], many of whom are in receipt of government funds. This is a marked departure from previous times when the sector was a grouping of charitable organisations. Service delivery fragmentation on the scale we see in Australia has resulted in repeated calls for tighter integration mechanisms, common language in understanding social issues and systems better organised in the interests of more efficient and effective responses to the types of social health issues alluded to from the data in this paper. Social quality theory may well offer such integrative utility.

## Conclusions

Social quality theory is an innovative and beneficial theoretical tool that warrants greater utilisation in research and policy development in social determinants of health. The findings presented in this paper provide only an example of how the survey conducted may be used to inform policy. The findings presented here are too general but provide a snapshot of the utility of the tool for policy development. This paper is a first attempt to demonstrate the operationalisation of social quality theory and how data outcomes may be used to inform policy. Statistical analyses revealed that people on lower incomes (less that $45000) had lower socio-economic security, lower levels of membership of organisations (lower social cohesion), experienced higher levels of discrimination and were involved in less political action (lower social inclusion) and had lower social empowerment. The findings were more mixed in terms of age, with people over 65 years experiencing lower socio-economic security, but having higher levels of social cohesion, experiencing lower levels of discrimination (high social inclusion) and engaging in more political action (higher social empowerment). In terms of gender, women had higher social cohesion than men, although also experienced more discrimination (lower social inclusion).

As demonstrated in this paper, the social quality approach keeps the social analytical lens wide, both in how the social is conceived and related, and in how social and health policy responses are formulated. In addition, it may be used to measure the outcomes of policy and political interventions over time through repeated measurement within a given population. We suggest that whilst we know the potential impact of policy on health in terms of the social and structural determinants, we need a way of measuring the affects of policy on improving the condition under which health is determined. We argue that the social quality approach is a comprehensive measure of social quality (as opposed to either piecemeal approaches, or approaches based on individual quality of life measures) which may be used as such a tool for monitoring the impact of policy on health. These data may then be used as a point of comparison for future investigations into the social quality of Australians as a means of identifying the potential effectiveness of public health policy.

## Competing interests

The authors declare that they have no competing interests.

## Authors' contributions

CNL and SBM participated in the design of the study, data collection, and piloting. TKG was responsible for the sampling strategy and obtaining a random sample of the electronic white pages. TKG, CNL, SBM and PRW carried out the data analysis. PRW, SBM and FV drafted the manuscript. PRW wrote the grant application and received funding for the study. All authors read and approved the final manuscript.

## Pre-publication history

The pre-publication history for this paper can be accessed here:

http://www.biomedcentral.com/1471-2458/11/630/prepub

## Supplementary Material

Additional file 1 **Social Quality questionnaire**. This is a copy of the validated questionnaire used within the study to measure Social Quality. Click here for file
