# Supplementary material for: Complex problems require complex solutions: the utility of social quality theory for addressing the Social Determinants of Health
Source: BMC Public Health. 2011 Aug 5;11:630. doi: 10.1186/1471-2458-11-630 (PMC3167771; doi:10.1186/1471-2458-11-630)
Supplement: Additional file 1 — Social Quality questionnaire. This is a copy of the validated questionnaire used within the study to measure Social Quality. [file 1471-2458-11-630-S1.PDF]

Social Quality of Life in Australia Survey

- YOUR ANSWERS WILL BE STRICTLY CONFIDENTIAL. You will not be able to be identified in any report from this study.

- Please answer each question according to the directions outlined by the question.

- Once completed, please return to Flinders University in the prepaid envelope provided.

Please contact paul.ward@flinders.edu.au to ask for author permission before reproducing the questionnaire

In the following questions please tick one box unless otherwise specified. In this case, you'll be provided with directions.

General Questions About You

Please indicate your **gender**.

☐ Male ☐ Female

In what year were you born? ______

What is your **religion**?

☐ No religion
☐ Buddhist
☐ Protestant
☐ Catholic
☐ Muslim
☐ Hindu
☐ Other (write in):__________
☐ Orthodox

What is your **marital status**?

☐ Never married
☐ Married
☐ Divorced
☐ Widowed
☐ Separated
☐ Defacto

Which **ONE** of the following **best** describes your current employment situations?

☐ Working full-time for pay
☐ Working part-time for pay
☐ Self-employed
☐ Working without pay in a family/other business
☐ Unemployed, looking for work
☐ Retired
☐ Full-time student
☐ Household duties not looking for paid work
☐ Not working because of a disability
☐ Other (please specify)

What mode of transport do you rely on the **most**?

☐ Personal Car
☐ Public Transport
☐ Bike
☐ Other (please specify)

Do you ever experience difficulty in using **public transportation**? (such as bus, subway, and train; not including taxi)

☐ Yes ☐ No ☐ Don't know

What is your **main source** of income? Please tick only one box.

☐ Wages or salaries
☐ Income from self-employment or farming
☐ Pension (retirement)
☐ Child benefit
☐ Unemployment, disability or any other social benefits
☐ Other income (e.g. from savings, property or stocks, etc.)

Please contact paul.ward@flinders.edu.au to ask for author permission before reproducing the questionnaire

Did you vote in the **last** general election?

☐ Yes ☐ No ☐ Not Eligible to vote

What was the **total annual income** received by everyone in your household BEFORE TAX in the last financial year?

Per year	
0 - $14,999	☐
$15,000 - $29,999	☐
$30,000 - $44,999	☐
$45,000 - $59,999	☐
$60,000 - $74,999	☐
$75,000 - $89,999	☐
$90,000 - $104,999	☐
$105,000 - $119,999	☐
$120,000 - $134,999	☐
$135,000 - $149,999	☐
$150,000 or more	☐
Don't know	☐

How **well** do you read English?

☐ Very well
☐ Quite well
☐ Not very well
☐ Not at all

Questions About Your Household

Which of the following living arrangements **best** describes your **household**?

☐ Live alone
☐ Live with partner/spouse only
☐ Live with partner/spouse and children
☐ Sole parent with children
☐ Live with parents or other related adults
☐ Live with other unrelated adults
☐ Other (please specify):________________

How **satisfied** are you with your financial situation?

**Completely dissatisfied** 1	2	3	4	5	6	7 **Completely satisfied**
☐	☐	☐	☐	☐	☐	☐

Please contact paul.ward@flinders.edu.au to ask for author permission before reproducing the questionnaire

During the past year, did you…

☐ 'Save money'
☐ 'Just get by'
☐ 'Spent some savings'
☐ 'Spent savings and borrowed money'

Have you received any of the following types of income over the past **12 months**? You can tick more than one box.

	**Yes**	**No**
Wages or salaries	☐	☐
Income from self-employment or farming	☐	☐
Pension (retirement)	☐	☐
Child benefit	☐	☐
Unemployment, disability or any other social benefits	☐	☐
Other income (e.g. from savings, property or stocks, etc.)	☐	☐
Informal support from family or relatives	☐	☐

How many children do you have? ___________

Questions About Your Life

Could you tell me how **important** each of these are in your quality of life?

	**Important**	**A little important**	**Not at all important**
A good education	☐	☐	☐
A good job	☐	☐	☐
A good standard of living	☐	☐	☐
Good accommodation	☐	☐	☐
A good family life	☐	☐	☐
Good health	☐	☐	☐

How **happy or unhappy** would you say you are with the following?

	**Very Happy**	**Happy**	**Average**	**Unhappy**	**Very Unhappy**
Your job	☐	☐	☐	☐	☐
Your standard of living	☐	☐	☐	☐	☐
Your accommodation	☐	☐	☐	☐	☐
Your family life	☐	☐	☐	☐	☐
Your health	☐	☐	☐	☐	☐
Your social life	☐	☐	☐	☐	☐

Please contact paul.ward@flinders.edu.au to ask for author permission before reproducing the questionnaire

Please indicate whether you or your **family** have experienced any of the following in the **last 12 months**.

	**Yes**	**No**
Costly medical expenses (such as hospitalization, operation, nursing home etc.)	☐	☐
Job loss or business bankruptcy	☐	☐
Job insecurity (such as getting switched from a regular to non-regular position)	☐	☐
Work injury	☐	☐
Becoming a victim of crime (such as fraud, robbery etc.)	☐	☐
Investment loss (such as share market/real estates, etc.)	☐	☐

How **often** do you have direct contact with…

	**Every day or almost every day**	**At least once a week**	**Once or twice a month**	**Several times a year**	**Less often**	**Don't have any**
Friends	☐	☐	☐	☐	☐	☐
Colleagues	☐	☐	☐	☐	☐	☐
Neighbours	☐	☐	☐	☐	☐	☐

How **often** do you do each of the following activities in your free time?

	**Daily**	**Several times a week**	**Several times a month**	**Several times a year**	**Never**
Watch TV, DVD, videos	☐	☐	☐	☐	☐
Go to live theatre	☐	☐	☐	☐	☐
Go to music concerts	☐	☐	☐	☐	☐
Go to live sport	☐	☐	☐	☐	☐
Go to museums or cultural heritage	☐	☐	☐	☐	☐
Go to the cinema	☐	☐	☐	☐	☐

For each of the following organisations, please indicate your **membership status**.

	**Don't belong**	**Member**
Church or religious organization	☐	☐
Sport or recreational organization	☐	☐
Art, music, educational, or cultural organization	☐	☐
Other community-based organization	☐	☐

In relation to your immediate **neighbourhood**, how **satisfied or unsatisfied** are you with each of the following?

	**Very unsatisfied**	**Somewhat unsatisfied**	**Somewhat satisfied**	**Very satisfied**
Level of noise	☐	☐	☐	☐
Level of air pollution	☐	☐	☐	☐
Access to recreational or green areas	☐	☐	☐	☐
Level of crime	☐	☐	☐	☐
Amount of rubbish in the streets	☐	☐	☐	☐

Please contact paul.ward@flinders.edu.au to ask for author permission before reproducing the questionnaire

During the past **12 months**, have you ever experienced **discrimination against you** due to any of the following reasons? You can tick more than one box.

Reason	Yes	No	Don't know
Physical/mental disability	☐	☐	☐
Age	☐	☐	☐
Sexual harassment	☐	☐	☐
Gender	☐	☐	☐
Nationality	☐	☐	☐
Physical appearance	☐	☐	☐
Ethnic background	☐	☐	☐
Criminal record	☐	☐	☐
Religion	☐	☐	☐
Other (Specify:___________________________)	☐	☐	☐

Questions About Your Health

On the **last occasion** you needed to see a doctor or medical specialist, to what extent did each of the following factors make it **difficult** for you to do so?

	Very difficult	A little difficult	Not difficult at all	Not applicable / never needed to see doctor
Distance to doctor's office / hospital / medical centre	☐	☐	☐	☐
Delay in getting appointment	☐	☐	☐	☐
Waiting time to see doctor on day of appointment	☐	☐	☐	☐
Cost of seeing the doctor	☐	☐	☐	☐

In general, would you say your **health** is...

☐ Very good
☐ Good
☐ Fair
☐ Bad
☐ Very bad

Do you have any **chronic (long-standing)** physical or mental health problem, illness or disability?

☐ Yes
☐ No

How long have you been seeing your current general practitioner or family physician?

☐ Less than one year
☐ 1 to 5 years
☐ 6-10 years
☐ Over 10 years
☐ I do not see a general practitioner (GP)

Please contact paul.ward@flinders.edu.au to ask for author permission before reproducing the questionnaire

If you had a **health problem** that needed **immediate** attention and your usual doctor was not available, how much would the following factors influence your decision to **trust a doctor** you have never seen before? Please circle a number from 1 to 3 or tick the box on the far right.

	A lot	Somewhat	Not at all	Do not know
The way they are dressed	☐	☐	☐	☐
They are wearing a white coat	☐	☐	☐	☐
They seem to be caring	☐	☐	☐	☐
They appear to be competent in their ability as a doctor	☐	☐	☐	☐
They appear to be older than 40	☐	☐	☐	☐
They appear to be younger than 40	☐	☐	☐	☐
They are female	☐	☐	☐	☐
They are male	☐	☐	☐	☐

Questions About Your Views on Others

Generally speaking, would you say that **most people** can be **trusted**?

- ☐ Yes
- ☐ No
- ☐ Don't know

Do you think that **most people** would **take advantage** of you if they had the chance?

- ☐ Yes
- ☐ No
- ☐ Have not thought about it

How much do you **trust** various **groups of people**?

	Trust them completely	Trust them somewhat	Do not trust them very much	Do not trust them at all	Have not thought about it	Not relevant
Your family	☐	☐	☐	☐	☐	☐
Your neighbours	☐	☐	☐	☐	☐	☐
People you meet for the first time	☐	☐	☐	☐	☐	☐
Your regular doctor	☐	☐	☐	☐	☐	☐
Doctors in general	☐	☐	☐	☐	☐	☐
A doctor you are seeing for the first time	☐	☐	☐	☐	☐	☐
People of another religion	☐	☐	☐	☐	☐	☐
People of another nationality	☐	☐	☐	☐	☐	☐
National political leader	☐	☐	☐	☐	☐	☐
Your local politician	☐	☐	☐	☐	☐	☐
Police officers	☐	☐	☐	☐	☐	☐

Please contact paul.ward@flinders.edu.au to ask for author permission before reproducing the questionnaire

How much do you **trust** the following **organisations** or **institutions**?

	Trust them completely	Trust them somewhat	Do not trust them very much	Do not trust them at all	Have not thought about it	Not relevant
Religious organisations	☐	☐	☐	☐	☐	☐
The press	☐	☐	☐	☐	☐	☐
The legal system	☐	☐	☐	☐	☐	☐
The media	☐	☐	☐	☐	☐	☐
Your government	☐	☐	☐	☐	☐	☐
United Nations	☐	☐	☐	☐	☐	☐
Banks	☐	☐	☐	☐	☐	☐

Have you ever **doubted** information from the following **organisations/institutions**?

	Yes	No	Have not received information from this organisation/institution
Your national government	☐	☐	☐
Credit card companies	☐	☐	☐
The media	☐	☐	☐

Have you ever **doubted** information from the following individual(s)?

	Yes	No	Have not received information from this individual
Your family doctor	☐	☐	☐
Doctors in general	☐	☐	☐
Family member	☐	☐	☐
Friends/people you know personally	☐	☐	☐

Have you ever requested a **second opinion** after receiving medical advice from a **doctor**?

- ☐ Yes
- ☐ No

How often do you **trust** your **government** to do what is in the best interest of their citizens?

- ☐ Almost always
- ☐ Some of the time
- ☐ Almost never
- ☐ Have not thought about it

Questions About Your Personal Views

How **interested** would you say you are in politics? Are you…

☐ Very interested ☐ Somewhat interested
☐ Not very interested ☐ Not at all interested

Please rank the following 4 items listed below from 1-4 regarding what you think the most **important aims** should be for Australia in the next 10 years (with 1 being the most important and 4 being the least important).

	Rank (1-4)
Making sure Australia has strong defence forces	
Making sure that people have more say in how things are done in their communities	
Cleaning up and protecting the environment	
A high level of economic growth	

Please contact paul.ward@flinders.edu.au to ask for author permission before reproducing the questionnaire

How **proud** are you to be an Australian? (If you do not identify as an Australian, please tick the last box.

☐ Very proud
☐ Quite proud
☐ Not very proud
☐ Not at all proud
☐ I do not identify as an Australian

When people **immigrate** to Australia, which one of the following do you think the government should do?

☐ Let anyone come who wants to
☐ Let people come as long as there are jobs available
☐ Put strict limits on the number of foreigners who can come here to work
☐ Prohibit people coming here to work
☐ Don't know

Please rate your view on the following scales.

**Incomes should be Made more equal**	1	2	3	4	5	6	**We need larger income differences as incentives for individual effort** 7
	☐	☐	☐	☐	☐	☐	☐
**The government should take more responsibility to ensure that everyone is provided for**	1	2	3	4	5	6	**People should take more responsibility to provide for themselves** 7
	☐	☐	☐	☐	☐	☐	☐

To what extent do you feel that you have a **sense of belonging** or **not belonging** as a member of the following…

	**Very close**	**Close**	**Distant**	**Very Distant**
Your neighbourhood	☐	☐	☐	☐
Your city/town	☐	☐	☐	☐
Your state	☐	☐	☐	☐
Australia	☐	☐	☐	☐
A World Citizen	☐	☐	☐	☐

How much do you **agree or disagree** with the following statements about men and women?

	**Strongly Agree**	**Agree**	**Neither agree nor disagree**	**Disagree**	**Strongly Disagree**	**Can't choose**
On the whole, men make better political leaders than women.	☐	☐	☐	☐	☐	☐
A university education is more important for a girl than for a boy	☐	☐	☐	☐	☐	☐
On the whole, women make better business executives than men.	☐	☐	☐	☐	☐	☐

Please contact paul.ward@flinders.edu.au to ask for author permission before reproducing the questionnaire

To what extent do you **agree or disagree** with the following statement? ‘Without trade unions, the working conditions of employees would be much worse than they are.’

☐ Strongly Agree
☐ Agree
☐ Neither agree nor disagree
☐ Disagree
☐ Strongly Disagree

Please indicate how **important** each of the following are in **your life**.

	Very important	Rather important	Not very important	Not at all important	Not applicable
Family	☐	☐	☐	☐	☐
Friends	☐	☐	☐	☐	☐
Respect for parents	☐	☐	☐	☐	☐
Politics	☐	☐	☐	☐	☐
Religion	☐	☐	☐	☐	☐

In your opinion how much **tension** is there between each of the following groups in Australia?

	A lot of tension	A bit of tension	No tension	Don’t know
Poor and rich people	☐	☐	☐	☐
Management and employees	☐	☐	☐	☐
Men and women	☐	☐	☐	☐
Old people and young people	☐	☐	☐	☐
Different ethnic groups	☐	☐	☐	☐
Different religious groups	☐	☐	☐	☐

Have you, or would you, **participate** in any of the political actions listed below?

	Have done	Might do	Would never do
Petition	☐	☐	☐
Boycotts	☐	☐	☐
Protests	☐	☐	☐
Strikes	☐	☐	☐
Online political actions	☐	☐	☐

Please contact paul.ward@flinders.edu.au to ask for author permission before reproducing the questionnaire

Please rate how strongly you **agree/disagree** with each of the following statements below:

	**Strongly Agree**	**Agree**	**Neither agree nor disagree**	**Disagree**	**Strongly Disagree**	**Can't choose**
I am optimistic about the future	☐	☐	☐	☐	☐	☐
In order to get ahead nowadays you are forced to do things that are not appropriate	☐	☐	☐	☐	☐	☐
I feel left out of society	☐	☐	☐	☐	☐	☐
Life has become so complicated today that I almost can't find my way	☐	☐	☐	☐	☐	☐
I don't feel the value of what I do is recognised by others	☐	☐	☐	☐	☐	☐

In **Australia**, how often do you think the following occur?

	**Always 1**	**2**	**3**	**4**	**5**	**6**	**Never 7**
That politicians take into account the views of citizens before making decisions.	☐	☐	☐	☐	☐	☐	☐
Business take into account the interests of citizens	☐	☐	☐	☐	☐	☐	☐

How **likely or unlikely** do you think that people can achieve a higher social status by their own effort?

- ☐ Very likely
- ☐ A little likely
- ☐ Neither likely nor unlikely
- ☐ A little unlikely
- ☐ Very unlikely
- ☐ Don't know

Thank you very much for completing this questionnaire.

Please return the questionnaire in the envelope provided (*no postage stamp required*)

Please contact paul.ward@flinders.edu.au to ask for author permission before reproducing the questionnaire
